# The Modified Early Warning Score as a Predictive Tool During Unplanned Surgical Intensive Care Unit Admission

**DOI:** 10.31486/toj.19.0057

**Published:** 2020

**Authors:** Annandita Kumar, Hussam Ghabra, Fiona Winterbottom, Michael Townsend, Philip Boysen, Bobby D. Nossaman

**Affiliations:** ^1^ University of Queensland Faculty of Medicine, Ochsner Clinical School, New Orleans, LA; ^2^ Department of Anesthesiology, Ochsner Clinic Foundation, New Orleans, LA; ^3^ Department of Pulmonary/Critical Care, Ochsner Clinic Foundation, New Orleans, LA; ^4^ Department of Surgery, Ochsner Clinic Foundation, New Orleans, LA; ^5^ Department of Anesthesiology, University of Mississippi School of Medicine, Jackson, MS

**Keywords:** *Clinical deterioration*, *critical care*, *early warning score*, *mortality*, *predictive value of tests*

## Abstract

**Background:** The Modified Early Warning Score (MEWS) has been proposed to warn healthcare providers of potentially serious adverse events. We evaluated this scoring system during unplanned escalation of care in hospitalized surgical patients during a 1-year period.

**Methods:** Following institutional review board approval, all consecutive, unplanned surgical admissions into the surgical intensive care unit (SICU) during 2016 were entered into this study. MEWS and patient demographics during bedside evaluation for SICU admission were extracted from electronic medical records. Logistic regression was used to analyze the association of MEWS with the incidence of future mortality. *P* values were set at <0.01 for statistical significance.

**Results:** In this series of 263 consecutive patients, the incidence of mortality following unplanned escalation of care was 29.3% (confidence interval [CI] 24.1% to 35.0%), ranging from 22% to 57%, with all positive MEWS values. The association of MEWS with future mortality was not statistically significant (*P*=0.0107). A misclassification rate of 0.29 (CI 0.24 to 0.35) was observed with this association.

**Conclusion:** MEWS provided no clinical benefit as an early warning system, as mortality was elevated throughout the MEWS scale in this clinical setting. The high misclassification rate indicates MEWS does not provide discriminatory support for patients at risk for mortality.

## INTRODUCTION

The Modified Early Warning Score (MEWS) has been proposed to warn healthcare providers of the potential development of serious adverse events, including unplanned escalation of care.^1-6^ MEWS is composed of bedside measurements of heart rate, respiratory rate, systolic blood pressure, temperature, and level of consciousness (alert, responsive to voice, responsive to pain, and unresponsive).^6,7^ The values of these measurements are scored and ranked, with a clinical response initiated once predetermined threshold scores are exceeded.^6,7^ Two studies have suggested that the use of early warning systems, such as MEWS, might be beneficial in reducing mortality in hospitalized patients.^8,9^ Pittard developed an outreach monitoring service in 3 surgical wards to assess the benefits of this new service on unplanned admissions to intensive care units (ICUs), length of stay, and mortality rates.^10^ However, McGaughey and colleagues expressed concerns that the implementation of the early warning system was not based upon robust, evidence-based research.^11^ Le Lagadec and Dwyer observed that although aggregated weighted scoring systems are frequently used, the efficiency of the specific early warning system appears to be dependent upon the patient cohort, facilities available, and staff training and attitude.^12^

We evaluated this scoring system when used as a component of bedside evaluation during unplanned escalation of care in hospitalized surgical patients following systemwide implementation of the electronic warning system available through the Epic electronic medical record.

## METHODS

Following institutional review board approval, all adult (≥18 years of age), consecutive, unplanned surgical admissions to the surgical intensive care unit (SICU) during 2016 were entered into this study. The dataset for MEWS analysis was 100% complete. Calculated MEWS values during bedside evaluation before unplanned SICU admission were extracted from electronic medical records. Data are expressed either as counts and percentages or as medians with 25%-75% interquartile and full ranges. Logistic regression was used to analyze the association of the bedside MEWS values on the incidence of future mortality. Key analyses have associated 95% confidence intervals (CI).^13,14^ The effect size for this model was analyzed with likelihood odds ratios.^15,16^ The discriminative ability of this model was analyzed with C-statistics.^17,18^ The predictive accuracy of this model was analyzed with misclassification rates.^19-21^ Internal model validation was conducted with the statistical technique of bootstrapping (1,000 cycles) to confirm that the calculated CIs provided a range of probable population values that were consistent with our data analysis in this clinical setting.^14,22,23^ *P* values were set for statistical significance at <0.01 to minimize the risk of false discovery rates or in declaring associations significant by chance alone.^24,25^ The program JMP 13.2 (SAS Institute) was used for the statistical analysis of the dataset.

## RESULTS

For this series of 263 consecutive patients, demographics and comorbidities during evaluation for unplanned escalation of care are shown in Table 1. The etiologies for unplanned SICU admission are shown in Table 2. The incidence of mortality following unplanned escalation of care was 29.3% (CI 24.1% to 35.0%). MEWS values ranging from 0 to 8, when plotted against future mortality rates, were not statistically significant (chi-square [χ^2^]=6.5, *P*=0.0107), with an unadjusted odds ratio per unit change of 1.2 (CI 1.1 to 1.5) and a C-index value of 0.60 (CI 0.54 to 0.66) (Figure).

**Table 1.** Demographics and Comorbidities in Patients With Unplanned Escalation of Care

Bedside Variable	All Patients n=263
Age, years, median [IQR]	61 [50-71]
Sex, male	144 (55)
Body mass index, kg/m^2^, median [IQR]	26.6 [23.1-33.3]
Comorbidities	
Systemic hypertension	103 (39.2)
Coronary artery disease	44 (16.7)
History of myocardial infarction	10 (3.8)
Nonsinus dysrhythmias	40 (15.2)
Coronary artery bypass graft	11 (4.2)
Congestive heart failure	46 (17.5)
Peripheral vascular disease	56 (21.3)
Tobacco abuse	9 (3.4)
Chronic obstructive pulmonary disease	20 (7.6)
Reactive airway disease	11 (4.2)
History of cancer	24 (9.1)
Diabetes	65 (24.7)
Chronic liver disease	47 (17.9)
Chronic renal insufficiency	49 (18.6)

Note: Data are shown as counts (%) unless otherwise indicated; IQR, interquartile range, 25%-75%.

**Table 2.** Admission Etiologies in Patients With Unplanned Escalation of Care

Etiology	Percentage of Patients n=263
Acute lung injury	33.2
Multiple organ dysfunction syndrome	22.8
Gastrointestinal insufficiency	17.2
Myocardial dysfunction	9.5
Vascular insufficiency	6.5
Acute tubular necrosis	3.0
Airway edema	2.2
Postoperative delirium	2.2
Pancreatitis	1.3
Hemorrhage	0.9
Wound infection	0.9
Splenic injury	0.4

**Figure.**
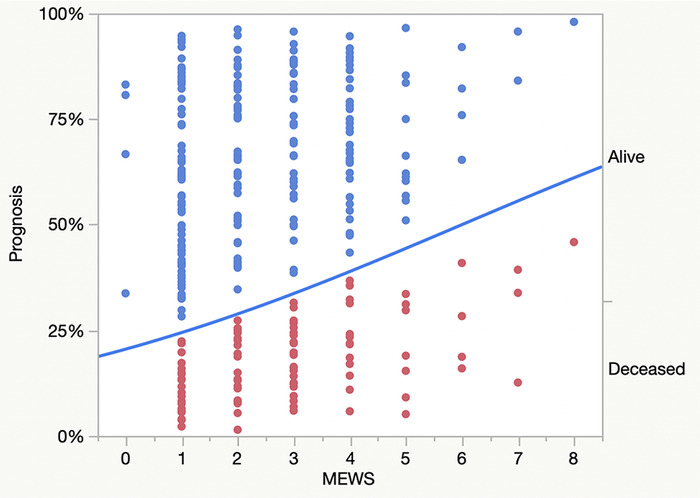
**Association of Modified Early Warning Scores (MEWS) on prognosis during unplanned escalation of care. The line plots the probability of prognosis by MEWS values. Points below the line identify deceased patients. Points above the line identify alive patients. The whole-model statistic is χ^2^=6.5, *P*=0.0107; C-index=0.60 (confidence interval [CI] 0.54 to 0.66). Following bootstrapping of the model (1,000 cycles), the whole-model statistic was within the CI range (0.56 to 18.3) of probable population values for this clinical setting**.

Probabilities and associated predictive modeling calculations across the range of MEWS values are shown in Table 3. Probability for mortality rates were observed with all positive MEWS values and ranged from 22% to 57% (Figure and Table 3). Large numbers of false positive and false negative values were observed with all MEWS values. A cut-point of 3 was calculated by the statistical program based upon highest Sensitivity – (1–Specificity) percentile (Table 3).

**Table 3.** Probabilities, Associated Calculations, and Cross-Classifications for Testing Across Modified Early Warning Scores (MEWS) in Patients With Unplanned Escalation of Care

MEWS	Probability for Mortality, %	1–Specificity, %	Sensitivity, %	Sensitivity – (1–Specificity), %	True Positive, n	True Negative, n	False Positive, n	False Negative, n
8	56.8	0.5	1.3	0.8	1	185	1	76
7	51.5	1.6	5.2	3.6	4	183	3	73
6	46.0	3.8	9.1	5.3	7	179	7	70
5	40.7	8.6	11.7	3.1	9	170	16	68
4	35.6	25.3	29.9	4.6	23	139	47	54
3	30.8	39.3	57.1	17.8*	44	113	73	33
2	26.3	64.5	81.8	17.3	63	66	120	14
1	22.3	97.9	100	2.1	77	4	182	0
0	18.8	100	100	0.0	77	0	186	0

Note: A cut-point of 3 was calculated in this model based upon the highest percentile value in the Sensitivity – (1–Specificity) column.

We developed a confusion matrix with our dataset to analyze the performance of this early warning system in which the true outcomes are known (Table 4). This model had an accuracy of 0.711 (CI 0.69 to 0.73), with a prevalence for survival of 0.707 (71%, CI 0.65 to 0.76) and a prevalence for fatality of 0.293 (29%, CI 0.241 to 0.35). The sensitivity of the model was 0.984 (CI 0.97 to 0.996), with a specificity of 0.052 (CI 0.019 to 0.080). The positive predictive value was 0.715 (CI 0.71 to 0.72), and the negative predictive value was 0.571 (CI 0.20 to 0.88). The likelihood odds ratio for a positive test was 1.038 (CI 0.99 to 1.08), and the likelihood odds ratio for a negative test was 0.310 (CI 0.06 to 1.61). The number needed to diagnose was 27.9 (CI 13.23 to 88.03), and the number needed to misdiagnose was 3.46 (CI 3.24 to 3.67). The kappa value was 0.049 (CI –0.015 to 0.103), and the Youden J value was 0.036 (CI –0.01 to 0.08). Finally, the misclassification rate observed in this study was 0.289 (29%, CI 0.27 to 0.31) (Table 4). Bootstrapping of this model calculated that the whole-model statistic (χ^2^=6.5) was within the CI range (0.56 to 18.3) of probable population values for this clinical setting.

**Table 4.** Confusion Matrix for Modified Early Warning Score During Bedside Evaluation in Unplanned Escalation of Care

	Actual Prognosis		
Predicted Prognosis	Alive	Deceased	Totals
Alive	183 (a or TP)	73 (b or FP)	256 (r1)
Deceased	3 (c or FN)	4 (d or TN)	7 (r2)
**Totals**	186 (c1)	77 (c2)	263 (t)

ARR, Absolute risk reduction; CI, confidence interval; DP, difference in proportions; FN, false negative; FP, false positive; TN, true negative; TP, true positive.

**Prevalence**=Alive [c1/t]=186/263=0.707 (71%) (CI 0.65 to 0.76); Deceased [c2/t]=77/263=0.293 (29%) (CI 0.241 to 0.35)

**Kappa**=0.049 (CI –0.015 to 0.103).

***Test statistics not dependent upon prevalence.***

**Sensitivity**=a/c1=183/186=0.984 (CI 0.97 to 0.996)

**Specificity**=d/c2=4/77=0.052 (CI 0.019 to 0.080)

**Positive predictive value**=a/r1=183/256=0.715 (CI 0.71 to 0.72)

**Negative predictive value**=d/r2=4/7=0.571 (CI 0.20 to 0.88)

**Positive likelihood ratio**=Sensitivity/(1–Specificity)=0.984/(1–0.052)=1.038 (CI 0.99 to 1.08)

**Negative likelihood ratio**=(1-Sensitivity)/Specificity=(1–0.984)/0.052=0.310 (CI 0.06 to 1.61)

**Odds ratio**=(a/b)/(c/d)=(183/73)/(3/4)=3.34 (CI 0.61 to 19.4)

**Relative risk**=(a/r1)/(c/r2)=(183/256)/(3/7)=1.67 (CI 0.89 to 6.08)

**Diagnostic odds ratio**=[Sensitivity/(1–Sensitivity)]/[(1–Specificity)/Specificity=[0.984/(1–0.984)]/[(1–0.052)/0.052]=3.373 (CI 0.61 to 19.36)

**Error odds ratio**=[Sensitivity/(1–Sensitivity)]/[Specificity/(1–Specificity)]=(0.984/[1-0.984])/(0.052/[1-0.052])=1,139 (CI 1,711 to 2,553)

**Difference in proportions**=[(a/r1) – (c/r2)]=[(183/256) – (3/7)]=0.286 (CI –0.09 to 0.60)

**Number needed to treat**=(1/absolute value of DP) which is equal to (1/absolute value of ARR)=1/0.286=3.49 (CI 1.66 to infinite)

**Absolute risk reduction**=[(c/r2) – (a/r1)]=[(3/7) – (183/256)]=which is equal to –DP=–0.286 (CI –0.60 to 0.09)

**Relative risk reduction**=[ARR/(c/r2)]=[–0.286/(3/7)]=–0.668 (CI –5.079 to 0.114)

**Youden J value**=(Sensitivity+Specificity–1)=(0.984+0.052–1)=0.036 (CI –0.01 to 0.08)

**Number needed to diagnose**=which is equal to (1/Youden J)=(1/0.036)=27.9 (CI 13.23 to 88.03)

***Test statistics dependent upon prevalence.***

**Accuracy**=(a+d)/t)=(183+4)/263=0.711 (71%) (CI 0.69 to 0.73)

**Misclassification rate**=[(c+b)/t]=(3+73)/263=0.289 (29%) (CI 0.27 to 0.31)

**Number needed to misdiagnose**=[1/(1–Accuracy)]=[1/(1–0.711)]=3.46 (CI 3.24 to 3.67)

## DISCUSSION

### Modified Early Warning Score

Unplanned SICU admission contributes to morbidity and mortality.^26^ The incidence of mortality following unplanned escalation of care in this study was 29.3% (CI 24.1% to 35.0%). This observation is similar to the reported incidences of mortality ranging from 17% to 76% in clinical studies of unplanned escalation of care.^26-31^

MEWS was originally proposed as a tool to inform healthcare providers of the potential for development of critical illness in emergency rooms and in high-care units, but it quickly became adopted for use on hospital wards.^2-4,6,7^ Subsequent studies have evaluated the benefits of early warning systems such as MEWS but have reported conflicting responses, with some studies showing benefits,^7,32^ whereas other studies have not shown beneficial effects.^33^ Cuthbertson et al observed that some physiologic measures and MEWS were predictive in surgical patients requiring ICU admission, but they acknowledged that their study was limited by missing data and that MEWS required prospective validation.^32^ In our study, increasing MEWS values were clinically, but not statistically, associated with prognosis.

As this study may be the first to contain a complete dataset in this clinical setting, we conducted internal validation with bootstrapping to determine probable population values,^14,22,23^ but our findings need external validation. The kappa and Youden J values were close to zero, suggesting that MEWS as an early warning system was unusable. In addition, the misclassification rate of 29% suggests poor calibration using MEWS as a tool in predicting mortality.^19-21^ Within our clinical setting, MEWS was not useful as an early warning system, and our findings suggest that an unmeasured confounder exists that triggered nursing services to notify the rapid response team.

Early studies identified that predictability with MEWS may have been limited because of incomplete datasets and inadequate healthcare personnel education.^1,2,34^ Ludikhuize et al were able to improve nursing identification of deteriorating patients following introduction of MEWS but noted that this early warning system was rarely used.^34^ However, our institution provided nursing education during development and implementation of MEWS, and this scoring system automatically, rather than manually, calculated and recorded scores in the electronic medical record with a color-coded alert system displayed on computers or smartphones for all patient healthcare providers. In the study by Ludikhuize et al, the need to manually calculate scores may have played a role in low adherence with MEWS.^34^

In our study, the statistical program calculated the optimum cut-point value of 3, and using this cut-point, we calculated an unadjusted odds ratio of 2.1 (CI 1.2 to 3.5) and a relative risk probability for death of 1.7 (CI 1.1 to 2.4) in patients with MEWS ≥3 compared to patients with MEWS values <3 (whole-model statistic χ^2^=7.0, *P*=0.0080), with a misclassification rate of 0.40 (CI 0.35 to 0.46). Although our results show that increasing mortality rates are associated with increasing MEWS values (Figure and Table 3), the key finding in our study is an unacceptable misclassification rate of 29%, wherein our group of patients with MEWS values of 1 and 2 had unacceptable mortality rates. We find that MEWS is an unacceptable predictive tool under escalation-of-care conditions.

### Statistical Analysis

In predictive modeling, forecasting adverse events is highly desirable when the potential prognosis is severe or if consequences increase with delayed diagnosis.^21^ The discriminative power of a model can be calculated by several mathematical processes to assess predictive accuracy.^21,35^ Sensitivity and specificity calculations provide estimates of illness probability, and predictive values provide additional assessments that patients with a positive test do have the condition or patients with a negative test do not have the condition (Table 4). The use of various odds ratios, especially the use of likelihood odds ratios, provides a measure of effect size (Table 4), and the use of C-statistics (Figure) provides a measure of discrimination.^15-18,35^ However, these test statistics may not perform well in low-prevalence conditions^7,36,37^ and may overestimate their benefits or underestimate the costs of clinical resources.^21,35,38^ Clinicians need a testing tool to limit the potential for negative consequences on patient health and on medical care expenditures.^35^ Misclassification rates support that answer (Table 3). Misclassification rates identify how often the model is wrong and account for the prevalence of the condition in question.^19-21^ In our study, the high misclassification rate for MEWS and the number needed to misdiagnose of 1 in 3.46 (Table 4) strongly suggest that this scoring system, when evaluated under these clinical conditions, is not effective as an early warning system.^19-21^

### Limitations and Strengths

One limitation of this study is that the dataset is not representative of all patients on general wards but of a clinical setting during an unplanned escalation of care, so these results should only be interpreted in this setting. Another limitation is the need for these results to be confirmed by other centers under similar clinical settings. However, as already noted, this model underwent bootstrapping to provide internal validation to allow inferences about similar clinical populations and to check the stability of the results.

One strength of this study is the robust, complete set of MEWS values from bedside evaluation of patients during escalation of care, the time when MEWS should be most useful. Measures of effect size and C-statistics for discriminative ability were provided, in which these values were poor. The major strength of this study is the use of misclassification rates, a valuable mathematical calculation that identifies when a tool incorrectly classifies patients, leading to misjudgments in clinical care.

## CONCLUSION

The key finding in this study is that MEWS is not an effective early warning system in surgical patients undergoing bedside evaluation during escalation of care. The misclassification rate in this model is high, and hence, MEWS could serve to misinform clinicians as to the nature of the patient's condition, with the result of either undertreatments, leading to severe consequences, or overtreatments, with attendant risks from unnecessary therapies and associated costs. Further, MEWS did not provide discriminatory support for patients at risk for mortality.

## Notes

This article meets the Accreditation Council for Graduate Medical Education and the American Board of Medical Specialties Maintenance of Certification competencies for Patient Care, Medical Knowledge, and Practice-Based Learning and Improvement.
